# Engineering a modular ^44^Ti/^44^Sc generator: eluate evaluation in preclinical models and estimation of human radiation dosimetry

**DOI:** 10.1186/s13550-023-00968-5

**Published:** 2023-02-28

**Authors:** Nadia Benabdallah, Hanwen Zhang, Ryan Unnerstall, Amanda Fears, Lucy Summer, Michael Fassbender, Buck E. Rodgers, Diane Abou, Valery Radchenko, Daniel L. J. Thorek

**Affiliations:** 1 Washington University School of Medicine, Mallinckrodt Institute of Radiology, 510 S. Kingshighway Boulevard, St. Louis, MO 63110 USA; 2 Program in Quantitative Molecular Therapeutics, Washington University School of Medicine, St. Louis, MO 63110 USA; 3 Washington University School of Medicine, Siteman Cancer Center, St. Louis, MO 63110 USA; 4 Chemistry Division, Los Alamos National Laboratory, PO Box 1663, Los Alamos, NM 87545 USA; 5 Department of Radiation Oncology, Washington University School of Medicine, St. Louis, MO 63110 USA; 6 Mallinckrodt Cyclotron Facility, Washington University School of Medicine, St. Louis, MO 63110 USA; 7 Life Sciences Division, TRIUMF, 4004 Wesbrook Mall, Vancouver, BC V6T 2A3 Canada; 8 Department of Chemistry, University of British Columbia, 2036 Main Mall, Vancouver, BC V6T 1Z1 Canada; 9 Department of Biomedical Engineering, Washington University, St. Louis, MO 63110 USA

**Keywords:** Isotope generator, Positron emission tomography, Scandium-44, Pharmacokinetics

## Abstract

**Background:**

^44^Sc/^47^Sc is an attractive theranostic pair for targeted in vivo positron emission tomographic (PET) imaging and beta-particle treatment of cancer. The ^44^Ti/^44^Sc generator allows daily onsite production of this diagnostic isotope, which may provide an attractive alternative for PET facilities that lack in-house irradiation capabilities. Early animal and patient studies have demonstrated the utility of ^44^Sc. In our current study, we built and evaluated a novel clinical-scale ^44^Ti/^44^Sc generator, explored the pharmacokinetic profiles of ^44^ScCl_3_, [^44^Sc]-citrate and [^44^Sc]-NODAGA (1,4,7-triazacyclononane,1-glutaric acid-4,7-acetic acid) in naïve mice, and estimated the radiation burden of ^44^ScCl_3_ in humans.

**Methods:**

^44^Ti/^44^Sc (101.2 MBq) in 6 M HCl solution was utilized to assemble a modular ZR resin containing generator. After assembly, ^44^Sc was eluted with 0.05 M HCl for further PET imaging and biodistribution studies in female Swiss Webster mice. Based on the biodistribution data, absorbed doses of ^44/47^ScCl_3_ in human adults were calculated for 18 organs and tissues using the IDAC-Dose software.

**Results:**

^44^Ti in 6 M HCl was loaded onto the organic resin generator with a yield of 99.97%. After loading and initial stabilization, ^44^ScCl_3_ was eluted with 0.05 M HCl in typical yields of 82.9 ± 5.3% (*N* = 16), which was normalized to the estimated generator capacity. Estimated generator capacity was computed based on elution time interval and the total amount of ^44^Ti loaded on the generator. Run in forward and reverse directions, the ^44^Sc/^44^Ti ratio from a primary column was significantly improved from 1038 ± 440 to 3557 ± 680 (Bq/Bq) when a secondary, replaceable, ZR resin cartridge was employed at the flow outlet. In vivo imaging and ex vivo distribution studies of the reversible modular generator for ^44^ScCl_3_, [^44^Sc]-citrate and [^44^Sc]-NODAGA show that free ^44^Sc remained in the circulation significantly longer than the chelated ^44^Sc. The dose estimation of ^44^ScCl_3_ reveals that the radiation burden is 0.146 mSv/MBq for a 70 kg adult male and 0.179 mSv/MBq for a 57 kg adult female. Liver, spleen and heart wall will receive the highest absorbed dose: 0.524, 0.502, and 0.303 mGy/MBq, respectively, for the adult male.

**Conclusions:**

A clinical-scale ^44^Ti/^44^Sc generator system with a modular design was developed to supply ^44^ScCl_3_ in 0.05 M HCl, which is suitable for further radiolabeling and in vivo use. Our data demonstrated that free ^44^ScCl_3_ remained in the circulation for extended periods, which resulted in approximately 10 times greater radiation burden than stably chelated ^44^Sc. Stable ^44^Sc/^47^Sc-complexation will be more favorable for in vivo use and for clinical utility.

**Supplementary Information:**

The online version contains supplementary material available at 10.1186/s13550-023-00968-5.

## Introduction

Positron emission tomography (PET) is well-established in oncologic radiological workflows to detect and monitor disease progression. The majority of investigations use the metabolic tracer ^18^F-fluorodeoxyglucose (^18^F-FDG). However, there is an increasing use of peptide ligands with radiometals for specific indications to delineate molecularly specific disease types. Replacement of positron emitters with therapeutic isotopes can then be used to localize cytotoxic treatments to sites of confirmed malignancy. This paradigm of radionuclide-based theranostics has attracted a great deal of research, clinical and pharmaceutical interest [1]. In particular ^68^Ga (*t*_1/2_ = 68 min, *E*_mean_ (*β*^+^) = 830 keV (89%)) and ^177^Lu (*t*_1/2_ = 6.72 d, *E*_mean_(*β*^−^) = 134 keV)-labeled somatostatin receptor peptides and prostate-specific membrane antigen inhibitors have been approved for PET imaging and treatment of neuroendocrine tumor and metastatic castration-resistant prostate cancer, respectively.

Among the investigated theranostic pairs [2], ^44^Sc/^47^Sc is well-suited for targeted in vivo PET and beta-particle treatment, respectively [3, 4]. ^44^Sc has a suitable half-life of 4.04 h for centralized radiopharmaceutical production along with highly abundant positron decay (*E*_mean_ (*β*^+^) = 632 keV (94%)) [5, 6]. ^47^Sc emits a low-energy *β*^−^ particle (*E*_mean_ (*β*^−^) = 162 keV) similar to ^177^Lu, with the potential for treating lesions with a half-life of 80.4 h. This is well-suited to the relatively fast pharmacokinetic profiles of small peptides [7].

^44^Sc can be obtained via either direction irradiation of natural or enriched calcium [8] or the decay of ^44^Ti (*t*_1/2_ = 60.6 ± 1.3 years) [4]. The potentially long utility of the ^44^Ti/^44^Sc generator system has many advantageous characteristics. It allows for daily elution over a long period of time, providing an attractive alternative for PET facilities that lack in-house cyclotron capabilities. The capability of ^44^Sc-labeled molecules have begun to be investigated in preclinical research. Multiple cancer xenografts models have been imaged, and several ^44^Sc-ligands have recently undergone initial clinical evaluation including [^44^Sc]-PSMA617 for imaging patients with metastatic prostate cancer [9, 10]. These in vivo studies have demonstrated that ^44^Sc-labeled ligands provide high contrast for disease delineation in pre-clinical xenografts and clinical patient studies.

In order to harness the potential for this isotope for theranostics, greater availability of the isotope and improved understanding of in vivo stability and pharmacodynamics is required [11–13]. In this work, we sought to produce and better understand how ^44^Sc is excreted in vivo, and to what degree this will impact the radiation burden in research and clinical use. To address these questions, we have engineered a clinical-scale ^44^Ti/^44^Sc generator using ZR resin [14]. Here, we use a reversible-flow modular column design with a disposable cartridge to recover any ^44^Ti breakthrough and have performed PET imaging and kinetic biodistribution studies with the eluted ^44^Sc material. These studies provide a comprehensive evaluation of the generator and produced material, including human dosimetry estimates for more widespread clinical use.

## Material and methods

All chemicals were obtained from commercial sources and were used without further purification. ^44^Ti/^44^Sc solution (111.0 MBq, 421.8 MBq/mg titanium) was obtained from Brookhaven National Laboratory, Department of Energy. Both ZR resin and 0.43-mL ZR resin cartridges were obtained from TRISKEM International. Radioactivity amounts of ^44^Sc were measured with a dose calibrator (CAPINTEC, CRC-15R) or a 2480 WIZARD^2^ automatic *γ*-counter (PerkinElmer). Radiochemical purity was analyzed with high-purity germanium gamma ray detector (HPGe, ORTEC, GEM-50195-S), and spectral acquisitions were acquired and analyzed by Gamma-Vision Software (version 8.0, Ametek). PEEK columns were obtained from VICI precision sampling, Inc for assembly of the ZR resin column. Deionized water (18.2 MΩcm, Rephile) and 99.999% trace-metal HCl (37 wt% in H_2_O) were used for preparation of ^44^Sc elution. Female Swiss Webster mice (6–8 weeks from Charlies River Laboratories) were purchased for in vivo pharmacokinetic studies. All radioactive material handling and animal experimentation were conducted in compliance with institutional regulations and approved by Environmental Health and Safety Radioactive Materials protocol #1169-01 and Institutional Animal Care and Use Committee protocol #22-0023.

### Design and assembly of ^44^Ti/^44^Sc generator

To construct the primary column approximately 200 mg of dry ZR resin was loaded in the PEEK column (50 × 4.0 mm), and the assembled ZR resin column was pre-conditioned with 3 × 2 mL of 6.0 M HCl. ^44^Ti/^44^Sc (101.2 MBq in 1.91 mL 6.0 M HCl) mixture. The initial washout solution was reloaded into the column, twice. After loading, two equivalently sized PEEK columns (pre-conditioned with 2 mL of 0.05 M HCl were attached to each end of the primary column to assemble the ^44^Ti/^44^Sc generator. ^44^Sc was eluted from the ^44^Ti/^44^Sc generator with 4 mL of 0.05 M HCl (flow rate:1 mL/min via syringe pump), and the elution profile was monitored in situ with a radiodetector (*γ*-RAM, IN/US) and recorded with Laura software (Lablogic). The radiochemical purity of the collected ^44^Sc was measured with HPGe and *γ*-counter immediately after elution, and 3 days later. Measurements with the γ-counter used an energy window of 430–580 keV for ^44^Sc, and 50–230 keV for ^44^Ti. Since the presence of ^44^Sc may significantly influence the measurement accuracy of ^44^Ti, aliquots of the elutions were stored for several days to afford time for ^44^Sc decay in order to calculate the ratio of ^44^Sc/^44^Ti in the eluted solution.

### Preparation of free and chelated ^44^Sc-NODAGA or Citrate for in vivo evaluation

After approximately 74 MBq of ^44^Sc was eluted into a vial with 4 mL of 0.05 M HCl solution, the ^44^Sc solution was adjusted to pH 6–7 with 2 M Na_2_CO_3_ (or 4 M NaOH) to prepare the ^44^ScCl_3_ solution. For preparation of ^44^Sc-citrate, sodium citrate (10 μL, 38.7 mM) was added to the eluted ^44^ScCl_3_ solution; and the mixture was incubated at 97 °C for 10 min. ^44^Sc-NODAGA was prepared with a similar procedure, except that the pH of the ^44^Sc solution was further adjusted with 1.0 M of ammonium acetate adjusted to pH 5 for chelation with NODAGA (10 μL, 13.6 mM). After incubation and cooling down to room temperature, ^44^Sc-citrate or ^44^Sc-NODAGA was prepared in a 30G syringe for in vivo administration.

### PET imaging of animals with free and chelated ^44^Sc (NODAGA, Citrate)

Female Naïve Swiss Webster mice (*N* = 4) were injected with 3.7 MBq/400 μL of ^44^ScCl_3_ (or ^44^Sc-NODAGA, or ^44^Sc-citrate) via tail vein catheterization under 2% isoflurane anesthesia. PET imaging was performed for an initial 0.5-h on-camera dynamic image acquisition, and for 10 min static scans at 1-, 2-, and 4-h post-injection using a microPET R4 rodent scanner (Siemens). The imaged mouse was centered in the field of view and maintained under 1–2% isoflurane anesthesia during PET imaging. The calibration factor of the PET scanner was determined with a mouse-sized phantom composed of a cylinder uniformly filled with an aqueous solution of ^18^F with a known activity concentration. Acquisitions were recorded using an energy window of 350–700 keV and coincidence-timing window of 6 ns. PET image data were corrected for detector non-uniformity, deadtime, random coincidences and physical decay and images were reconstructed by an iterative 3D maximum a priori algorithm.

The acquired PET images were analyzed using ASIPro software (Siemens). Volume of interest (VOI) analysis of the acquired images was performed using ASIPro software, and the observed value (percent injected activity/cubic centimeter, %IA/cc) represents the mean radiotracer accumulation in the organs. The sequential radioactivity measurements (%IA/cc) were plotted over time post-administration.

### Kinetic biodistribution of ^44^ScCl_3_ in naïve mice

Animals were administered 3.7 MBq/400 μL of ^44^ScCl_3_ for kinetic biodistribution studies. Four animals at each time point 5, 30, 60, 120, 240 and 1440 min post-injection were submitted for CO_2_ asphyxiation prior to tissue dissection. The organs of interest were collected, rinsed of excess blood, blotted, weighed, and counted with a 2480 WIZARD^2^ automatic *γ*-counter. We computed the percent of injected activity per gram of tissue (%IA/g) by normalizing the activity of each tissue to an injection standard, and the sample mass.

### Estimation of human radiation dose

Biodistribution data of ^44^ScCl_3_ in the Naïve Swiss Webster mice were extrapolated to human organs using the relative organ mass scaling method [15–17]. In this method, the animal organ data reported as percent of injected activity per gram of organ, $\left( {\frac{{\% {\text{IA}}}}{{{\text{g}}_{{{\text{organ}}}} }}} \right)_{{{\text{mouse}}}}$, is extrapolated using the animal and human whole-body masses, ${\mathrm{kg}}_{\mathrm{TBweight}}$, and the human organs masses, $\left({\mathrm{g}}_{\mathrm{organ}}\right)_{\mathrm{human}}$, employing the following equation:

$$\left( {\frac{{\% {\text{IA}}}}{{{\text{organ}}}}} \right)_{{{\text{human}}}} = \left[ {\left( {\frac{{\% {\text{IA}}}}{{{\text{g}}_{{{\text{organ}}}} }}} \right)_{{{\text{mouse}}}} \times \left( {{\text{kg}}_{{\text{TBweight}}} } \right)_{{{\text{mouse}}}} } \right] \times \left( {\frac{{{\text{g}}_{{\text{organ}}} }}{{{\text{kg}}_{{{\text{TBweight}}}} }}} \right)_{{\text{human}}}$$

The human organs masses were used as defined for adult male and female in the IDAC Dose 2.1 application [18]. This scaling was not applied to the organs of the gastrointestinal tract. Organ integrated time-activity were determined by numerical integration of time activity data. The cumulative activity, Ã, between time 0 and the first measured time point was calculated assuming a linear increase from 0 to the first measured activity. The Ã between the first measured time point and the last measured time point was integrated numerically using trapezoidal approximation. The Ã from the last measured time point to infinity was integrated considering only the physical decay. It was assumed that the radioisotope does not relocate following the last imaging point. For walled organs (heart, large intestine, small intestine, and stomach), the residence time was assigned entirely to the organ walls; with the large intestine, the residence time was divided evenly between the right and left colons. The bone residence time was likewise evenly divided between cortical and trabecular bone [19].

The cumulated activities for each organ were then used to compute the absorbed doses by IDAC Dose 2.1 [18]. The mean normal-organ absorbed doses (mGy/MBq administered) and the effective dose (mSv/MBq administered) for ^44^ScCl_3_ were calculated for standard human adults (female and male). Additionally, the biodistribution data of ^44^ScCl_3_ were used to model the absorbed doses for ^47^ScCl_3._ Time activity curves representing ^47^ScCl_3_ were calculated, taking into account the different half-life of the modeled radionuclide.

### Statistical analysis

Data calculated using Microsoft Excel are expressed as mean ± SD. Student’s unpaired t test (GraphPad Prism 9) was used to determine statistical significance at the 95% confidence level. Differences with *p* values < 0.05 were considered to be statistically significant.

## Results

### Design, assembly and performance of a dual-direction multicolumn ^44^Ti/^44^Sc generator

**Fig. 1:**
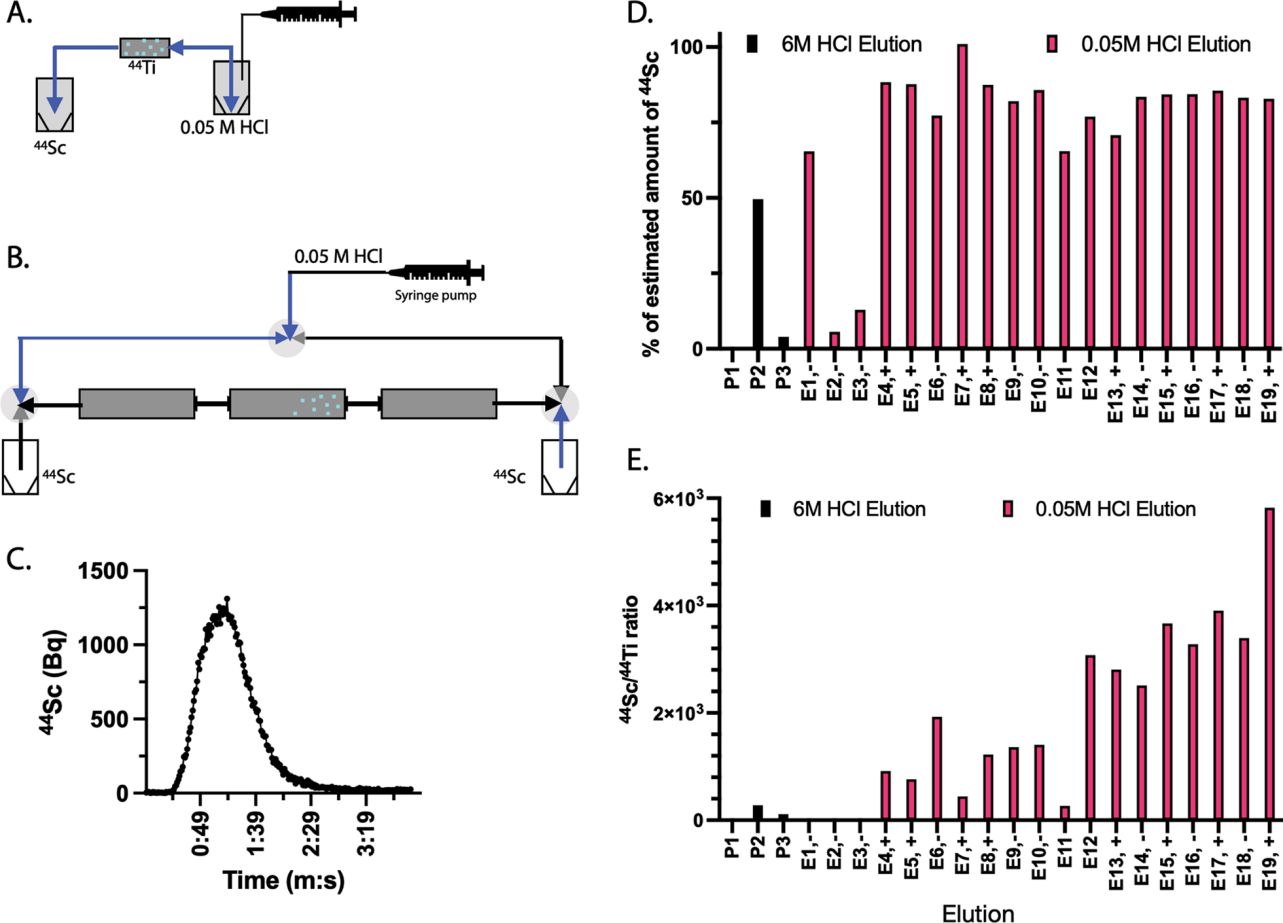
^44^Ti/^44^Sc generator and ^44^Sc elution. **A** Pilot micro-scale generator with uni-directional elution; **B** Clinical-scale modular generator with bidirectional elution; **C** Elution profiles of ^44^Sc with a mobile phase of 0.05 M HCl at a flow rate of 1.0 mL/min, which was monitored in situ with in-line gamma detector; **D** A consistent amount of ^44^Sc activity is collected after loading to the modular generator; **E** ^44^Sc/^44^Ti ratio in the eluted ^44^Sc solution. E4 to E11 elution is bidirectional without additional ZR resin cartridge; E12 to E19 elution is bidirectional with an additional ZR resin cartridge

Initial studies were performed to determine the ZR resin capacity for loading and retaining ^44/nat^Ti using a single-direction flow design (Fig. 1A). We determined that 25 mg of dry ZR resin is able to trap 23 μg of titanium. The provided material was specified as containing 87.7 μg/mCi of ^44/nat^Ti, indicating approximately 80.32 μg/mCi of ^nat^Ti in excess of carrier-free ^44^Ti. We observed significant breakthrough of ^44^Ti from this initial generator after loading, with approximately 20% of the loaded ^44/nat^Ti was washed out from the micro-scale pilot generator through the first ten uses of this (one-way elution) system. Thus, a modular clinical-scale generator system was pursued. Here, ^44^Ti loaded on a primary column is further sequestered on additional columns in each flow direction, and ^44^Sc was eluted with 0.05 M HCl using an alternating bidirectional flow to minimize migration and loss of the parent isotope. Here, the direction of liquid flow is changed after each run. Figure 1B depicts the loading of ^44^Ti onto modular column generator and final assembly.

The primary (central) column of the clinical-scale generator system comprised of 200 mg ZR resin, loaded in a PEEK column with size of 50 × 4.0 mm containing 101.2 MBq (2.736 mCi) of ^44^Ti in 6.0 M HCl in 1.91 mL (Fig. 1B). The loading was performed by pumping of the 6 M HCl ^44^Ti/^44^Sc solution through the column. After loading the pass-through solution onto the column twice, 99.97% of the ^44^Ti was absorbed on the column, with 35 kBq detected in further passed through (measured after decay of the daughter). With ^44^Ti loaded on the primary column, two PEEK columns filled with 200 mg ZR resin were assembled at both terminals (Fig. 1B).

A lower concentration of HCl (0.05 M) was used to elute ^44^Sc (Fig. 1C) as it is amenable for radiopharmaceutical preparations. The initial first three column elutions were performed on the same day in the same direction with 0.05 M of HCl. As ^44^Ti was loaded under high concentration conditions of 6 M HCl, the shift to 0.05 M of HCl to elute ^44^Sc created a transient resin condition, resulting in higher initial radioactivities of ^44^Ti release. As expected, each of these initial elutions released decreasing amounts of ^44^Ti: 3.43% (3474 kBq); 0.66% (651 kBq); 0.39% (381 kBq). These values are significantly greater than those compared to the ^44^Ti breakthrough at later use (elution E4 and further; Fig. 1D). After transition and stabilization, ^44^Sc was eluted bidirectionally with 1 mL/min of 0.05 M HCl for 4 min to generate ^44^ScCl_3_ in a yield of 82.9 ± 5.3% (*N* = 16, elution sample: E4-E19), which was normalized to the estimated generator capacity. Estimated generator capacity is computed based upon the total amount of ^44^Ti loaded on the generator, and the time interval between sequential elutions.

To simulate daily clinical-use conditions, the elution interval of E4 to E19 was approximately 24 h. The ^44^Sc/^44^Ti ratio obtained was 1038 ± 440 (*N* = 8) from E4 to E11. From elution E12 and on, an additional disposable ZR resin cartridge (0.3 mL) was employed at the flow outlet to further recover ^44^Ti breakthrough; ^44^Sc/^44^Ti ratios were then improved to 3557 ± 680 (*N* = 8; Fig. 1E).

### PET imaging of animals with free and chelated ^44^Sc

**Fig. 2:**
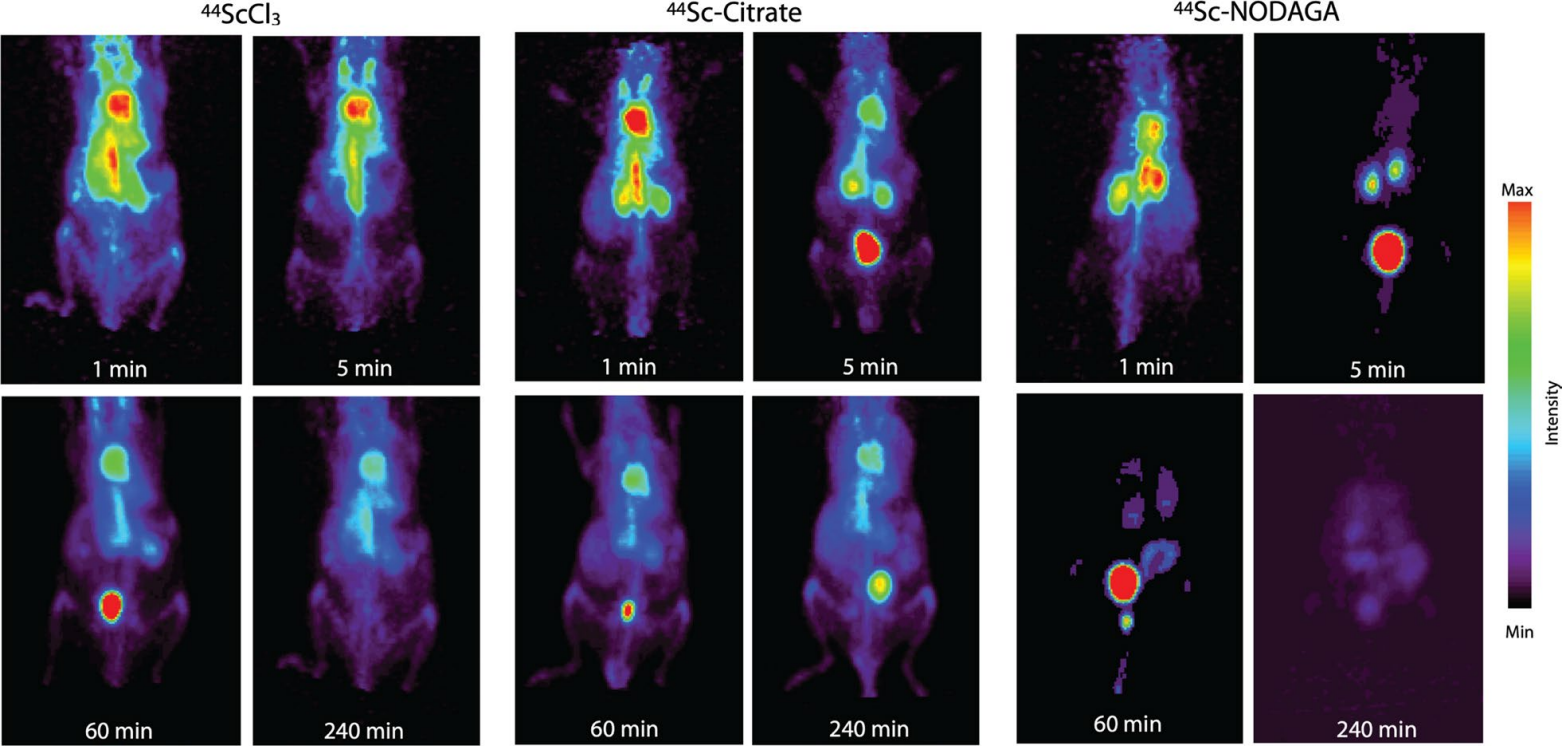
PET imaging of the in vivo distribution of ^44^ScCl_3_ and citrate or [^44^Sc]-NODAGA. Representative maximum intensity projections of the PET acquisitions at indicated times for each species. Later phase images reveal that ^44^Sc remained in circulation considerably longer than that of either chelated ^44^Sc formulations. The more stable [^44^Sc]-NODAGA is rapidly excreted via the urinary system

**Fig. 3:**
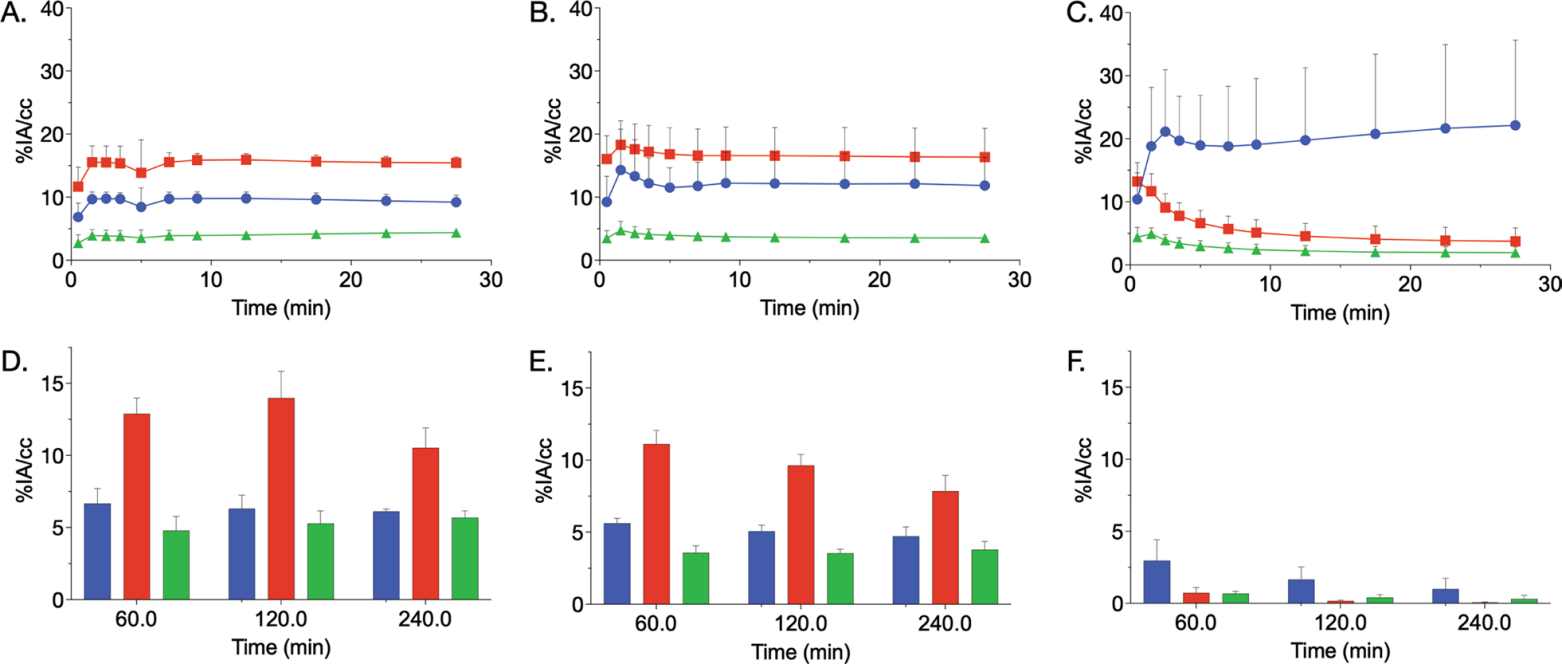
Quantitative analysis of PET imaging of ^44^ScCl_3_ and citrate or NODAGA chelated ^44^Sc. Volumes of interest (VOI) analysis of dynamic imaging of free ^44^Sc (**A**), [^44^Sc]-citrate (**B**) and [^44^Sc]-NODAGA (**C**). VOI analysis of static images of free ^44^Sc (**D**), [^44^Sc]-citrate (**E**) and [^44^Sc]-NODAGA (**F**). Legends: (Blue filled circle) Kidney, (orange filled square) Heart, (green filled triangle) Liver. Error bars represent the standard deviation from *N* ≥ 4 subjects

We next investigated the in vivo absorption, distribution and excretion of intravenously administered ^44^ScCl_3_ and chelated ^44^Sc (citrate and NODAGA). Dynamic PET imaging was performed initially on-camera (0.5 h) followed by static imaging at 1-, 2-, and 4-h post-injection. Representative acquisitions are shown in Fig. 2 and volume of interest analysis is presented in Fig. 3. PET imaging of ^44^ScCl_3_ showed significant cardiac signal in the acute post-injection phase, indicative of plasma binding and long circulation characteristics (Figs. 2 and 3). Within 1-h post-injection (Fig. 3A and D), renal accumulation of ^44^Sc was higher than that in the liver (*P* < 0.0001 at one half hour; 6.64 ± 0.84 vs 4.79 ± 0.81%IA/cc, *P* = 0.04 at 1 h), and there was no significant difference (Fig. 3D) at late time points (2 h: 6.31 ± 0.67 vs 5.28 ± 0.72%IA/cc, *P* = 0.16; 4 h: 6.11 ± 0.13 vs 5.68 ± 0.34%IA/cc, *P* = 0.14). A minor portion of ^44^Sc was eliminated via urinary excretion, resulting in visible bladder signal at 1-h post-administration (Fig. 2).

In contrast to free ^44^Sc ([^44^Sc]ScCl_3_), ^44^Sc complexed with citrate ([^44^Sc]-citrate) reveals a substantially higher kidney accumulation (Fig. 3B) out to 0.5-h post-injection (0.5 h: 9.21 ± 0.95 (^44^Sc) vs 11.85 ± 3.01%IA/cc ([^44^Sc]-citrate), *P* = 0.23), lower kidney accumulation at later time points (2 h: 6.31 ± 0.67 vs 5.07 ± 0.31%IA/cc, *P* = 0.05; 4 h: 6.11 ± 0.13 vs 4.72 ± 0.47%IA/cc, *P* = 0.006), and lower accumulation in the heart (2 h: 13.98 ± 1.52 vs 9.63 ± 0.63%IA/cc, *P* = 0.05; 4 h: 10.52 ± 1.16 vs 7.85 ± 0.81%IA/cc, *P* = 0.02) than that of free ^44^Sc (Fig. 3E). This indicates that the intact [^44^Sc]-citrate was rapidly excreted through the urinary system. The bladder was visible from 5-min to 4-h post-injection (Fig. 2). [^44^Sc]-citrate also displayed a lower accumulation in the liver than that of free ^44^Sc (Fig. 3D and 3E).

A stable chelation system was also evaluated. Here, [^44^Sc]-NODAGA demonstrated a short circulation in blood (*t*_1/2_ = 2.53 min with a range between 1.69 to 3.94 min), and the majority of the administered radiotracer was excreted into the bladder via the kidney within 5 min (Fig. 2). Kinetic PET imaging of ^44^Sc-NODAGA at 1-, 2-, and 4-h post-injection showed a lower and statistically significant accumulation in all organs evaluated in comparison with either free ^44^Sc or ^44^Sc-citrate (Fig. 3C and 3F; *P* < 0.05). However, ^44^Sc-NODAGA showed a varied accumulation in the kidneys within the 0.5-h post-injection (dynamic PET imaging), which resulted in mean renal values of uptake that are higher than that of free ^44^Sc and ^44^Sc-citrate (Fig. 3C).

### Biodistribution of free ^44^Sc in healthy mice

**Fig. 4:**
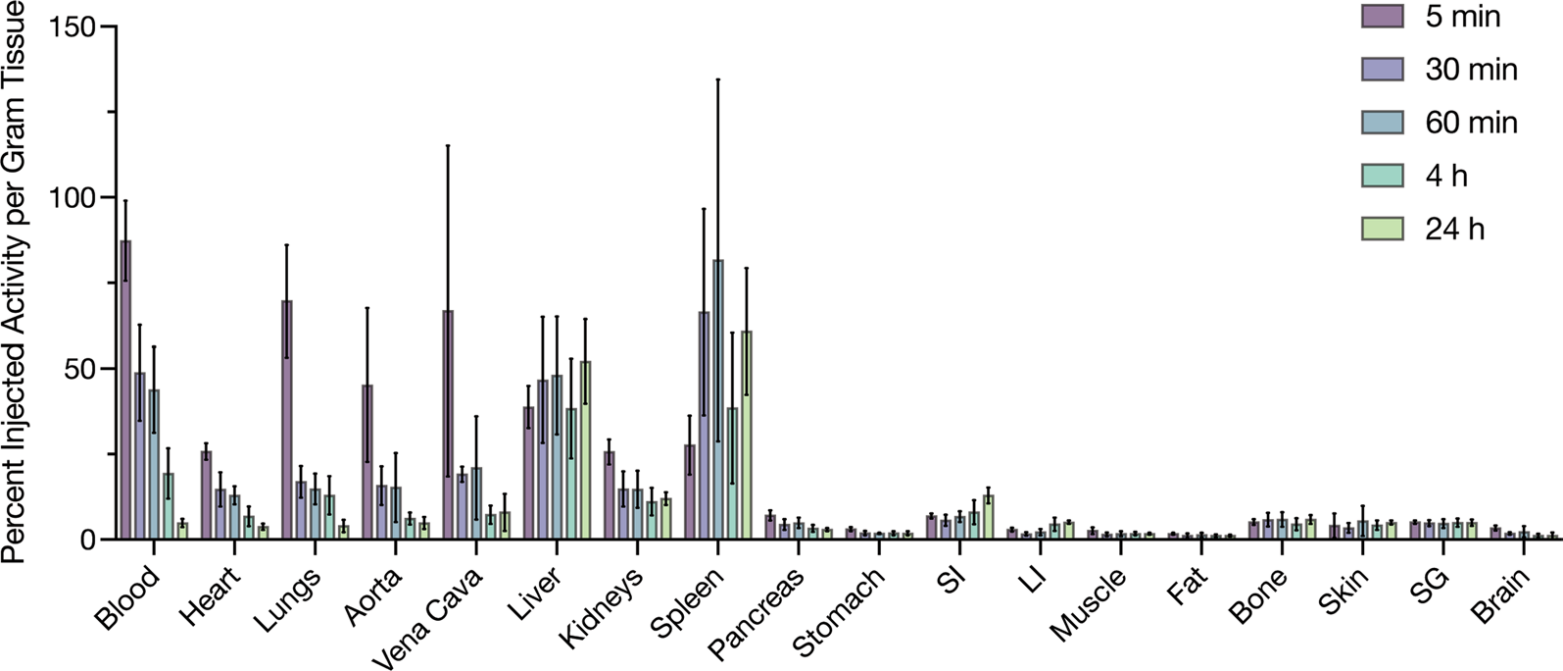
Kinetic distribution of ^44^ScCl_3_ in Swiss Webster mice. Free ^44^Sc (ScCl_3_) circulates in the blood with an effective half-lives of 2.0 min (rapid phase) and 133 min (slow phase), respectively, and is excreted from the body through both urinary and hepatic systems. Low accumulation in all other organs is observed at extended time points. Small Intestine (SI), Large Intestine (LI), and Salivary Glands (SG)

To further confirm the observation of free ^44^ScCl_3_ in mice, a kinetic biodistribution study was performed. Results are shown in Fig. 4, and percent injected activity per gram values for each organ are included as Additional file 1: Table S1. The ^44^ScCl_3_ is rapidly distributed in blood, heart, aorta, cava, lung, liver, kidneys and spleen. High activity levels in the blood, heart, aorta and vena cava confirm the persistence of ^44^Sc in blood from the PET imaging. Analysis of the excretion profile show effective half-lives of 2.0 min (rapid phase) and 133 min (slow phase), respectively. The free ^44^Sc was then excreted primarily through the liver, and a minor portion through the kidneys. An increased uptake was found in the spleen, 27.6 ± 7.3%IA/g at 5 min, and 60.8 ± 13.5%IA/g at 24 h-post-injection, and peak accumulation is at 1-h post-injection (81.6 ± 39.7%IA/g). Low background levels of accumulation were measured in all other collected organs, including pancreas, bone, brain, salivary glands, stomach, intestine, fat, skin, and muscle.

### Estimation of human radiation dose

The estimated absorbed dose was extrapolated from the female murine data, and the results are listed in Table 1. The effective dose for a 70-kg adult male was 0.146 and 0.179 mSv/MBq, and 0.310 and 0.369 mSv/MBq for female for ^44^ScCl_3_ and ^47^ScCl_3_, respectively. For example, it would be 16.2–19.9 mSv and 34.4–41.0 mSv from an intravenously injected radioactivity of 111 MBq (3 mCi) of ^44^ScCl_3_ and ^47^ScCl_3_, respectively. The absorbed doses for ^47^ScCl_3_ are higher than the absorbed doses for ^44^ScCl_3_, except for the adrenal, heart wall and red bone marrow. Among all organs, the liver, spleen, and heart wall received the highest absorbed dose: 0.524, 0.502, and 0.303 mGy/MBq for the adult male and ^44^ScCl_3_, respectively. The majority of organs in an adult female will receive higher absorbed dose than that of a male, excepting the breast and colon wall, which indicate that gender difference may be a factor in the irradiation burden.

**Table 1:** Absorbed doses per unit activity administered for ^44^ScCl_3_ and ^47^ScCl_3_ (mGy/MBq)

Organs	Absorbed Dose of ^44^ScCl_3_(mGy/MBq)		Absorbed Dose of ^47^ScCl_3_(mGy/MBq)	
	Male	Female	Male	Female
Adrenals	0.198	0.252	0.186	0.237
Brain	0.033	0.040	0.049	0.058
Breast	0.061	0.055	0.045	0.038
Colon wall	0.160	0.157	0.417	0.437
Endosteum (bone surface)	0.060	0.082	0.042	0.077
Gallbladder wall	0.277	0.297	0.303	0.336
Heart wall	0.303	0.361	0.249	0.294
Kidneys	0.244	0.299	0.496	0.601
Liver	0.524	0.615	1.680	2.030
Lung	0.240	0.276	0.229	0.263
Muscle	0.047	0.056	0.075	0.091
Pancreas	0.211	0.227	0.271	0.295
Red (active) bone marrow	0.124	0.174	0.080	0.111
Salivary glands	0.060	0.070	0.153	0.185
Skin	0.050	0.058	0.151	0.182
Small intestine wall	0.194	0.226	0.720	0.825
Spleen	0.502	0.596	1.850	2.260
Stomach wall	0.268	0.317	0.605	0.696
Effective dose 60 [mSv/MBq]	0.146	0.179	0.310	0.369

## Discussion

There is an increased interest in the development and implementation of theranostic nuclear medicine approaches for personalized patient management. Access to radioisotopes with desirable characteristics for quantitative PET imaging that are chemically analogous to therapeutic isotopes is an area of particular focus. Recent preclinical and clinical studies have investigated ^44^Sc-labeled small molecules as a promising positron-emitting diagnostic and surrogate for ^47^Sc-based radiotherapy. In comparison with gallium-68 (1.13 h), the half-life of ^44^Sc (3.97 h) affords advantages for labeling, quality control evaluation, transport logistics and the biokinetics of many tracers. The imaging characteristics for emissions from ^44^Sc have also been shown to be well-suited for delineation of small lesions [20] [21]. Cyclotron production of ^44^Sc through irradiation of natural calcium metal or liquid targets enables tertiary medical centers and large production facilities to produce the isotope [22] [6]. Alternatively, distributed generator systems that separate parent ^44^Ti from would enable on-site production. In this study, we built and evaluated a modular ^44^Ti/^44^Sc generator, and further investigated the absorption, distribution, and excretion of the activity after a single intravenous injection of generator eluate in female mice.

Consistent with prior investigation [4, 13, 14], ^44^Ti was efficiently loaded on the resin, and we observed that this material can re-distribute on the column following repeated ^44^Sc elutions. This resulted in breakthrough of ^44^Ti and has the potential to contaminate the radiopharmaceutical and work space for compounding. To avoid ^44^Ti breakthrough from the generator, a bidirectional elution approach has been employed to delay the breakthrough by others, including Filosofov et al. [23] and Radchenko et al. [14]. A bidirectional elution approach cannot prevent ^44^Ti redistribution and breakthrough completely, and the ^44^Ti/^44^Sc generator must be re-assembled after a period of use. Therefore, we engineered the modular clinical-scale generator to allow us to: (1) recover ^44^Ti efficiently and conveniently; (2) replace the columns independently; and (3) load ^44^Ti to the ZR resin generator semi-automatically with a minimum radiation dose to the operation personnel.

It has been reported that ^44^Ti has a consistent absorption efficiency on ZR resin across a wide range of HCl concentrations [14]. The ^44^Ti/^44^Sc stock solution was provided dissolved in 6 M HCl solution. We therefore loaded to the primary ZR resin column under the condition of 6.0 M HCl with ^44^Ti/^44^Sc. More than 99.9% of ^44^Ti was trapped. While ^44^Sc can be eluted efficiently with 4.0–6.0 M HCl, the high concentration of HCl here would complicate safe-handling and requires additional adjustment of pH conditions to reach suitable conditions for radiolabeling. Thus, a lower concentration of 0.05 M HCl was chosen to elute ^44^Sc. A significantly higher ^44^Ti breakthrough was observed in the first three elutions using this lower concentration. We put forward that ^44^Ti^3+^ may be hydrolyzed into ^44^Ti(OH)_x_ or ^44^TiO_2_ under the condition of less than 4 M HCl solution. During the transition from 6 M HCl to 0.05 M HCl, the absorbed ^44^Ti^3+^ may be quickly hydrolyzed and released from the resin. After elution with 0.05 M HCl for several days, ^44^Sc was obtained in a consistent yield with a high ^44^Sc/^44^Ti ratio. To further limit breakthrough of ^44^Ti from ^44^Sc elution, a disposable ZR resin cartridge (0.3 mL) was utilized at the flow outlet. Our results showed that ^44^Sc/^44^Ti ratio was further improved by 342%. Notably these small amounts of absorbed ^44^Ti on this disposable cartridge can be recovered by pass through of 6 M HCl/0.65% H_2_O_2_ [14], which can be combined, dried and redissolved in 0.05 M HCl solution for re-loading onto the center column of the generator.

PET imaging was performed to measure pharmacokinetic profiles of ^44^ScCl_3_ and chelated ^44^Sc with either citrate or NODAGA. After intravenously injection, dynamic PET imaging showed that free ^44^ScCl_3_ is distributed in blood (heart) and lung and that activity remained in circulation for an extended period. Ex vivo analyses recapitulated high radioactivity in the blood, heart, aorta and vena cava confirming ^44^Sc is mainly remained in blood with half-lives of 2.0 min (rapid phase) and 133 min (slow phase), respectively. Similar to ^68^GaCl_3_ PET imaging results in rats [24], ^44^ScCl_3_ in mice was slowly excreted through the kidney and liver. Our biodistribution results also show accumulation of ^44^ScCl_3_ in the spleen, with background levels of accumulation in all other tested organs. We hypothesize that similar to the Fe^+3^ and ^45^Ti [25] ion, a major portion of ^44^Sc^+3^ is bound to transferrin after intravenous administration. This results in extended circulation times and slow kinetics of excretion without specific accumulation in the heart wall or vasculature. Neither PET imaging nor biodistribution studies identified ^44^ScCl_3_ to be excreted via feces or the intestine. This is in contrast to the elimination of ^68^Ga or ^64^Cu, in which this gastrointestinal clearance presents a complication for interpreting preclinical imaging data. Together, these data motivate use of very high in vivo stability chelators for ^44/47^Sc targeted agents and for understanding of artifactual distributions.

^44^Sc-citrate showed a similar in vivo pharmacokinetic profile with an increased rate of clearance. The major difference identified was a higher kidney accumulation as ^44^Sc forms only a weak complex with citrate that may prevent rapid complexation by components in the blood. When the more stable ^44^Sc-NODAGA was used, activity was observed to transit into the bladder rapidly, which is consistent with the in vivo profiles of ^44^Sc-labeled peptides utilizing DOTA and NODAGA [26] or other novel chelate-conjugated ligands [27]. Further evaluation of the chemical identity of the generator output and its impact on radiotracer labeling and distribution will be conducted. This is of particular interest for future comparison of generator- and cyclotron-produced ^44^Sc.

Together, differences in distribution of free and chelated scandium imply that any unlabeled ^44^Sc/^47^Sc in the solution of ^44^Sc/^47^Sc-chelated ligand may cause a significant increase in the observed circulation time. To further clarify what amount of absorbed dose may be caused by the unlabeled ^44^Sc/^47^Sc, the data of a kinetic biodistribution in the mice was extrapolated to male and female adults. We observed a gender difference of irradiation dose and that the free-^44^Sc has a significantly higher irradiation burden than that of the conjugated ^44^Sc-ligands, as expected. For example, the mean effective dose of [^44^Sc]-PSMA617 in male patient is 0.0389 mSv/MBq [28], whereas the unconjugated ^44^Sc (current work) is 0.146 mSv/MBq. Similar results have been found in other critical organs, including spleen, liver and red marrow (0.185 vs 0.502; 0.107 vs 0.524; 0.0331 vs 0.124 mSv/MBq). These data indicate that a high purity for ^44/47^Sc-conjugated ligand is required for safe and effective radionuclide-based treatment in the future and provide insight into assessment of the radiation burden from decomplexed radioisotope in vivo.

## Conclusion

A clinical-scale ^44^Ti/^44^Sc generator system with a modular design was been developed which can supply over 74 MBq (2 mCi) of ^44^Sc in 4 mL of 0.05 M HCl. The generator has consistent performance characteristics, and the eluted material was evaluated in animal models for imaging and distribution studies. Our data demonstrated that free ^44^ScCl_3_ (unchelated) remained in the circulation for extended periods and was excreted predominantly through the liver and spleen, which resulted in a significant absorbed dose difference over stably-chelated ^44^Sc. Our results reveal that highly in vivo stable ^44^Sc/^47^Sc-complexation will be more favorable for successful translation and clinical utility.

## Supplementary Information

**Additional file 1**. Biodistribution of ^44^ScCl_3_ in healthy Swiss Webster mice. Description of data: Activity concentration per organ at time points after injection of ^44^ScCl_3_ in Naïve female mice.

## Data Availability

The datasets generated and analyzed during the current study are available upon direct request to the authors upon reasonable request.
