# Supplementary material for: Engineering a modular 44Ti/44Sc generator: eluate evaluation in preclinical models and estimation of human radiation dosimetry
Source: EJNMMI Res. 2023 Feb 28;13:17. doi: 10.1186/s13550-023-00968-5 (PMC9975127; doi:10.1186/s13550-023-00968-5)
Supplement: Supplementary file 1 — Additional file 1. Biodistribution of 44ScCl3 in healthy Swiss Webster mice. Description of data: Activity concentration per organ at time points after injection of 44ScCl3 in Naïve female mice. [file 13550_2023_968_MOESM1_ESM.docx]

**SUPPLEMENTAL MATERIAL**

**Title**:

Engineering a Modular ^44^Ti/^44^Sc Generator: Eluate Evaluation in Preclinical Models and Estimation of Human Radiation Dosimetry

**Authors:**

Nadia Benabdallah^1,2^, Hanwen Zhang^1,2,3,^*, Ryan Unnerstall^1^, Amanda Fears^1,2^, Lucy Summer^1^, Michael Fassbender^4^, Buck E. Rodgers^1,3,5^, Diane Abou^1,3,6^, Valery Radchenko^7,8^, Daniel LJ Thorek^1,2,3,9,^*

**Affiliations:**

1. Mallinckrodt Institute of Radiology, Washington University School of Medicine, St. Louis, Missouri, United States of America, 63110.

2. Program in Quantitative Molecular Therapeutics, Washington University School of Medicine, St. Louis, Missouri, United States of America, 63110.

3. Siteman Cancer Center, Washington University School of Medicine, St. Louis, Missouri, United States of America, 63110.

4. Chemistry Division, Los Alamos National Laboratory, PO Box 1663, Los Alamos, New Mexico United States of America, 87545.

5. Department of Radiation Oncology, Washington University School of Medicine, St. Louis, Missouri, United States of America, 63110.

6. Mallinckrodt Cyclotron Facility, Washington University School of Medicine, St. Louis, Missouri, United States of America, 63110.

7. Life Sciences Division, TRIUMF, 4004 Wesbrook Mall, Vancouver, BC, Canada, V6T 2A3.

8. Department of Chemistry, University of British Columbia, 2036 Main Mall, Vancouver, BC, Canada, V6T 1Z1.

9. Department of Biomedical Engineering, Washington University, St. Louis, Missouri, United States of America, 63110.

* Co-Corresponding Authors.

Dr. Hanwen Zhang and Dr. Daniel LJ Thorek

[hanwen.zhang@wustl.edu](mailto:hanwen.zhang@wustl.edu) [thorekd@wustl.edu](mailto:thorekd@wustl.edu)

Mallinckrodt Institute of Radiology

Washington University School of Medicine

510 S. Kingshighway Boulevard

St. Louis Missouri United States of America

63110

Supplemental Table 1. Biodistribution of ^44^ScCl_3_ in healthy Swiss Webster mice

| %IA/g  Tissues | ^44^ScCl_3_ | | | | |
| --- | --- | --- | --- | --- | --- |
|  | 5 min | 30 min | 60 min | 240 min | 1440 min |
| Blood | 87.3 ± 9.8 | 48.7 ± 12.0 | 43.8 ± 8.3 | 19.4 ± 5.7 | 4.87 ± 0.78 |
| Heart | 25.8 ± 1.9 | 14.7 ± 3.5 | 12.9 ± 2.1 | 6.80 ± 2.24 | 3.74 ± 0.75 |
| Lungs | 69.6 ± 12.2 | 16.9 ± 3.2 | 14.8 ± 3.6 | 13.0 ± 4.0 | 3.99± 1.26 |
| Aorta | 45.2 ± 16.3 | 15.8 ± 4.6 | 15.3 ± 8.4 | 6.15 ± 1.22 | 4.85 ± 1.27 |
| Vena Cava | 66.8 ± 40.8 | 19.1 ± 1.7 | 21.0 ± 11.0 | 7.29 ± 2.06 | 7.97 ± 3.93 |
| Liver | 38.8 ± 4.4 | 46.7 ± 15.6 | 48.0 ± 12.5 | 38.3 ± 10.5 | 52.1 ± 8.9 |
| Kidneys | 25.7 ± 3.1 | 14.8 ± 4.4 | 14.7 ± 4.0 | 11.1 ± 3.2 | 12.0 ± 1.4 |
| Spleen | 27.6 ± 7.4 | 66.5 ± 22.8 | 81.6 ± 40.0 | 38.4 ± 18.0 | 60.8 ± 13.5 |
| Pancreas | 7.09 ± 1.02 | 4.42 ± 1.18 | 4.91 ± 1.12 | 3.27 ± 0.80 | 2.91 ± 0.35 |
| Stomach | 3.07 ± 0.44 | 1.87 ± 0.54 | 1.83 ± 0.14 | 1.86 ± 0.48 | 1.82 ± 0.44 |
| Small Intestine | 6.91 ± 0.54 | 5.64 ± 1.37 | 6.73 ± 1.17 | 8.03 ± 2.83 | 12.9 ± 1.9 |
| Large Intestine | 2.90 ± 0.41 | 1.66 ± 0.37 | 2.17 ± 0.59 | 4.52 ± 1.54 | 5.06 ± 0.40 |
| Muscle | 2.60 ± 0.76 | 1.55 ± 0.50 | 1.74 ± 0.55 | 1.76 ± 0.35 | 1.73 ± 0.22 |
| Fat | 1.71 ± 0.23 | 1.12 ± 0.48 | 1.33 ± 0.50 | 1.12 ± 0.32 | 1.13 ± 0.24 |
| Bone | 5.14 ± 0.66 | 5.83 ± 1.38 | 5.88 ± 1.58 | 4.47 ± 1.24 | 5.86 ± 0.96 |
| Skin | 4.10 ± 2.65 | 3.42 ± 1.03 | 5.43 ± 3.16 | 4.24 ± 1.05 | 4.98 ± 0.46 |
| Salivary glands | 5.10 ± 0.37 | 4.84 ± 0.62 | 4.67 ± 0.96 | 4.94 ± 0.73 | 4.94 ± 0.69 |
| Brain | 3.39 ± 0.54 | 1.81 ± 0.26 | 2.31 ± 1.16 | 1.12 ± 0.46 | 1.11 ± 0.72 |
